# Virological outcomes with Bictegravir/Emtricitabine/Tenofovir alafenamide (B/F/TAF) in people previously treated with darunavir‐based antiretroviral therapy

**DOI:** 10.1111/hiv.70204

**Published:** 2026-02-01

**Authors:** Rhianna Sheridan, Yasmin Osei‐Kuffour Ekert, Lucy Campbell, Mark Zuckerman, Sally Hawkins, Kate Childs, Frank A. Post

**Affiliations:** ^1^ King's College Hospital NHS Foundation Trust London UK; ^2^ King's College London London UK

**Keywords:** adherence, Bictegravir, darunavir, HIV, resistance, virological failure

## Abstract

**Background:**

Darunavir‐based antiretroviral therapy (ART) is commonly used in people with HIV who experience adherence challenges and/or have complex resistance patterns. Changes in ART commissioning have led to an increased use of Bictegravir/Emtricitabine/Tenofovir alafenamide (B/F/TAF) in these populations despite limited real‐world outcome data.

**Methods:**

Single centre, retrospective analysis of virological outcomes in individuals previously treated with Darunavir who initiated B/F/TAF before 01/01/2025. Logistic regression was used to analyse associations with sustained virological suppression (HIV RNA <200 copies/mL) on B/F/TAF.

**Results:**

Of the 223 individuals who initiated B/F/TAF, 207 (median age 52 [40–58] years, 38% female, 69% Black ethnicity, 24% with CD4 < 200 cells/mm^3^ and 36% with HIV RNA ≥200 copies/mL) contributed virological outcome data. Over a median of 2.4 [1.3–3.3] years, 153 (74%) maintained or achieved sustained virological suppression, 11 (5.3%) had a single viral load ≥200 copies/mL and 43 (20.8%) experienced virological failure. Participants with CD4 < 200 cells/mm^3^ (aOR 0.15 [95%CI 0.07–0.33]) and HIV RNA ≥200 copies/mL (aOR 0.17 [0.08–0.34]) at B/F/TAF initiation were less likely to achieve sustained virological suppression; historical resistance‐associated mutations (RAMs) were not associated with virological outcome. Of the 32 participants successfully genotyped, 3 had novel INSTI mutations (E157Q, L74LM) and 4 had not previously documented NRTI mutations (M184V/I, D67DN, Y115F) mutations.

**Conclusions:**

Substituting of Darunavir‐based ART with B/F/TAF in this challenging population was associated with treatment‐emergent INSTI and NRTI resistance. Historical resistance did not predict virological outcomes and treatment‐emergent resistance did not preclude re‐suppression on B/F/TAF, suggesting that adherence remains a major barrier to achieving long‐term virological success.

## INTRODUCTION

Darunavir (DRV) is a protease inhibitor (PI) with a high genetic barrier to resistance; it is a preferred option for people with HIV who have developed virological failure with complex resistance profiles [1]. A second‐line antiretroviral therapy (ART) trial comparing DRV to dolutegravir (DTG), each coadministered with two nucleoside/nucleotide reverse‐transcriptase inhibitors (NRTI), showed non‐inferior suppression of HIV RNA through 96 weeks, albeit with emergent integrase strand‐transfer inhibitor (INSTI) resistance‐associated mutations (RAMs) occurring in a small proportion (4%) of participants in the DTG arm and no emergent PI RAMs in the DRV arm [2]. A further trial of DTG in people with HIV who failed non‐nucleoside reverse transcriptase inhibitor (NNRTI)‐based ART and most of whom had multiple NRTI RAMs reported treatment‐emergent INSTI RAMs in <1% of participants [3]. These studies confirm the preserved efficacy of DTG in the presence of extensive NRTI resistance.

Like DTG, Bictegravir is another second‐generation INSTI co‐formulated with emtricitabine and tenofovir alafenamide (B/F/TAF). In ART‐naïve individuals, the use of B/F/TAF achieved high levels of virological suppression, and no treatment‐emergent RAMs were reported during five years of follow‐up [4]. In a clinical trial of ART‐naïve individuals with advanced HIV disease, <1% of participants in the B/F/TAF arm developed resistance mutations over 48 weeks [5], while no or few (<1%) treatment‐emergent RAMs were encountered in real‐world cohorts of >1000 participants followed on B/F/TAF for up to 2 years [6, 7, 8]. Preserved efficacy of B/F/TAF in the presence of extensive NRTI resistance, including the K65R/N mutation, has been reported [9, 10, 11].

As B/F/TAF provides robust virological efficacy in the setting of both wild‐type and NRTI‐resistant HIV infection, a high genetic barrier to resistance and coverage for hepatitis B in a convenient single tablet regimen, B/F/TAF has increasingly replaced DRV‐based ART in individuals with suboptimal virological control, a history of virological failure, adherence challenges or complex resistance profiles. We evaluated virological outcomes in people with HIV transitioning from DRV‐based ART to B/F/TAF to assess the appropriateness of this change in practice.

## METHODS

The study was conducted as a service evaluation and received institutional approval, but as per UK regulations, did not require ethical review or informed consent.

We retrospectively identified all people with HIV at King's College Hospital who had ever been prescribed DRV‐based ART and B/F/TAF using electronic medical records. We included those who had previously been prescribed DRV (any DRV‐containing regimen) and who initiated B/F/TAF prior to 01‐January‐2025 (including both direct switches from DRV to B/F/TAF and switches from non‐DRV regimens to B/F/TAF in those previously treated with DRV); we excluded individuals who received B/F/TAF together with other antiretroviral agents or as part of end‐of‐life care, and those who had been prescribed but never initiated B/F/TAF. Our analyses focused on individuals with at least one HIV viral load on B/F/TAF in whom virological outcomes could be evaluated (Figure S1).

Demographic characteristics, HIV acquisition risk, ART history, CD4 cell counts, HIV viral loads and hepatitis co‐infection status were extracted from electronic medical records. Historical genotypic resistance data (Sanger sequences) were reviewed to identify NRTI, NNRTI, PI and INSTI RAMs and interpreted using the Stanford HIVDB algorithm [12]. In participants with viraemia on B/F/TAF, genotypic resistance profiles were abstracted or additionally performed on stored plasma samples where available and reviewed for treatment‐emergent RAMs when compared with historical profiles. Virological outcomes following the initiation of B/F/TAF were classified as follows: [1] sustained virological suppression, with all HIV RNA values <200 copies/mL during follow‐up; [2] unconfirmed viraemia, with a single HIV RNA ≥200 copies/mL after >3 months on B/F/TAF; and [3] virological failure, with >1 HIV RNA >200 copies/mL >3 months after B/F/TAF initiation; a cut‐off of 200 was chosen as measurements above this threshold are less likely to represent viral load blips and more likely to provide genotypic resistance test results, and all participants contributed a minimum follow‐up of 4 weeks.

Participant characteristics and virological outcomes were described. Logistic regression was used to analyse associations between a priori chosen variables (CD4 cell count, HIV RNA, archived resistance profiles and ART status [PI‐based regimen, PI‐sparing regimen, off ART]) at baseline and achieving sustained virological suppression on B/F/TAF. Multivariable models were adjusted for age (< or ≥ 35 years), sex and ethnicity (Black vs. White/other). All analyses were conducted using STATA v16.

## RESULTS

A total of 252 individuals who were previously managed on Darunavir were prescribed B/F/TAF. Of these, 29 were excluded for never starting B/F/TAF, receiving B/F/TAF with other antiretrovirals or initiating B/F/TAF in 2025 or as part of end‐of‐life care. Among the remaining 223 individuals, 16 discontinued B/F/TAF or did not have viral load measurements on B/F/TAF, leaving 207 participants with virological outcomes (Figure S1). The median age of the participants was 51.6 (IQR 40.3–58.1) years, 38% were female, and 69% identified as Black African or Caribbean. At the time of starting B/F/TAF, the median CD4 cell count was 388 (IQR 202–591) cells/mm^3^ and 133 (64%) had an HIV RNA <200 copies/mL (including 114 [55%] with HIV RNA <50 copies/mL). Historical resistance data were available for 185 (reverse‐transcriptase and protease) and 41 (integrase) participants, with NRTI RAMs documented in 29%, NNRTI RAMs in 39%, PI RAMs in 33% and INSTI RAMs in 22% (Table 1). Of the 54 participants with NRTI RAMs, 27 had M184V/I only, 19 had 1–2 TAMs (+/−M184V/I), 5 had 3–4 TAMs (+/−M184V/I), and 3 had K65R + M184V/I. Two participants had major INSTI RAMs (Tables 2 and S1).

**TABLE 1:** Characteristics at B/F/TAF initiation of the 207 participants included in the analysis, stratified by virological outcome.

Demographics		All	Sustained viral suppression (*N* = 153)	Viraemia (*N* = 54)
Age (years)	Median (IQR)	51.6 (40.3, 58.1)	52.1 (43.9, 59.0)	45.4 (24.7, 54.3)
Sex (female)	*N* (%)	79 (38)	52 (34)	27 (50)
Ethnicity (Black)	*N* (%)	142 (69)	100 (65)	42 (78)
HIV characteristics				
HIV risk				
MSM	*N* (%)	60 (29)	52 (34)	8 (15)
Heterosexual	*N* (%)	110 (53)	83 (54)	27 (50)
IVDU	*N* (%)	9 (4)	5 (3)	4 (7)
Vertical	*N* (%)	26 (13)	12 (8)	14 (26)
Other/unknown	*N* (%)	2 (1)	1 (1)	1 (2)
Time since HIV diagnosis	Median (IQR)	16.4 (10.2, 22.5)	16.7 (10.0, 22.8)	15.5 (11.1, 20.7)
Time since starting ART	Median (IQR)	13.1 (8.2, 20.2)	12.9 (8.0, 20.9)	13.2 (9.2, 18.6)
Current/recent CD4 cell count (cells/mm^3^)	Median (IQR)	388 (202, 591)	439 (259, 626)	207 (108, 386)
HIV RNA (log copies/mL)	Median (IQR)	1.5 (1.3, 3.8)	1.3 (1.3, 2.3)	3.9 (2.0, 4.8)
Hepatitis B (surface Ag positive)	*N* (%)	17 (8.3)	12 (8)	5 (9)
Hepatitis C (anti‐HCV positive)	*N* (%)	14 (6.8)	8 (5)	6 (11)
ART regimen and viral load				
ART‐regimen containing a PI*	*N*	149	117	32
CD4 cell count <200 (cells/mm^3^)	*N* (%)	25 (17)	13 (11)	12 (38)
HIV RNA ≥200 copies/mL	*N* (%)	37 (25)	21 (18)	16 (50)
ART‐regimen containing an INSTI/NNRTI	*N*	35	25	10
CD4 cell count <200 (cells/mm^3^)	*N* (%)	8 (23)	4 (16)	4 (40)
HIV RNA ≥200 copies/mL	*N* (%)	14 (40)	5 (20)	9 (90)
Off ART	*N*	23	11	12
CD4 cell count <200 (cells/mm^3^)	*N* (%)	16 (70)	6 (55)	10 (83)
HIV RNA ≥200 copies/mL	*N* (%)	23 (100)	11 (100)	12 (100)
HIV resistance (historical genotypes)				
PI resistance (any)	*N* (%)	61/185 (33)	46/133 (34)	15/52 (29)
NNRTI resistance (any)	*N* (%)	72/185 (39)	55/133 (41)	17/52 (33)
NRTI resistance (M184V/I, K65R, TAMs)	*N* (%)	53/185 (29)	41/133 (31)	12/52 (23)
INSTI resistance	*N* (%)	9/41 (22)	7/29 (24)	2/12 (17)

*Note*: *N* = 146 (98%) Darunavir, *N* = 3 (2%) Atazanavir.

Abbreviations: Ag, antigen; ART, antiretroviral therapy; B/F/TAF=Bictegravir/Emtricitabine/Tenofovir alafenamide; HCV, hepatitis C virus; INSTI, integrase strand‐transfer inhibitor; IVDU, intravenous drug use; MSM, men who have sex with men; NNRTI, non‐nucleoside reverse‐transcriptase inhibitor; NRTI, nucleoside/nucleotide reverse‐transcriptase inhibitor; PI, protease inhibitor.

**TABLE 2:** Virological outcomes on B/F/TAF.

Baseline resistance profile (based on historical genotypes)	*N*	Sustained virological suppression	Unconfirmed VL ≥200 copies/mL	Virological failure	Emergent resistance	
					NRTI^a^	INSTI^a^
Overall cohort	207	153 (74)	11 (5)	43 (21)	4/33	3/32
HIV RNA <200 copies/mL	133	116 (87)	7 (5)	10 (8)	0/9	1/9
HIV RNA ≥200 copies/mL	74	37 (50)	4 (5)	33 (45)	4/24	2/23
CD4 cell count ≥200 cells/mm^3^	158	130 (82)	8 (5)	20 (13)	3/15	3/16
CD4 cell count <200 cells/mm^3^	49	23 (47)	3 (6)	23 (47)	1/18	0/16
NRTI RAMs at or prior to baseline						
No tenofovir/emtricitabine RAMs*	132	92 (70)	9 (7)	31 (23)	3/23	2/22
M184V/I only**	27	20 (74)	0	7 (26)	1/6	1/6
1–2 TAMs (±M184V/I)***	19	14 (78)	1 (6)	3 (17)	0/4	0/4
3–4 TAMs (±‐M184V/I)****	5	5 (100)	0	0	0/0	0/0
K65R + M184V/I*****	3	2 (67)	0	1 (33)	0/0	0/0
Integrase RAMs at or prior to baseline						
No INSTI RAMs	32	22 (69)	2 (6)	8 (25)	0/7	0/6
Accessory INSTI RAMs^+^	7	5 (71)	0	2 (29)	0/2	0/2
Major INSTI RAMs^++^	2	2 (100)	0	0	0/0	0/0
No historical NRTI resistance profile	22	20 (91)	1 (5)	1 (5)	0/0	0/0
No historical INSTI resistance profile	166	124 (75)	9 (5)	33 (20)	3/24	3/24

*Note*: Date expressed as *N* (%), except emergent resistance (expressed as *N*/*N* with resistance data on B/F/TAF). TAMs: M41L, D67N, K70R, L210W, T215F/Y, K219Q/E/N; INSTI major: T66A/I/K, E92G/V/Q, G118R, F121C/Y, E138K/A/T, G140S/A/C/R, Y143K/G/S/A/C/R/H/S, P145S, Q146P/R/L, S147G, Q148H/K/R/N, V151L, N155H/S/T, R263K; INSTI accessory: H51Y, L74F/M, Q95K, T97A, A128T, G149A, V151A, S153Y/F, E157Q, G163R/K, S230R, D232N. Additional RAMs: * INSTI accessory (L74M, G140E, Q146H, V151I, E157Q, G163R; *N* = 5); ** NRTI K70E/Q/T or Y115F (*N* = 1), INSTI major (N155H; *N* = 1), INSTI accessory (G163R; *N* = 1); *** NRTI K70E/Q/T or Y115F (*N* = 2), INSTI accessory RAM (Q95K; *N* = 1), *N* = 9 had M184V/I; **** NRTI K70E/Q/T or Y115F (*N* = 1), INSTI major RAM (Q148K; *N* = 1), N = 5 had M184V/I; ***** *N* = 1 also had 1 TAM, *N* = 3 had M184V/I; ^+^ 2 TAMs + M184V/I (*N* = 1), M184V/I (*N* = 1); ^++^ 3TAMs + M184M/I (*N* = 1), M184V/I (*N* = 1).

Abbreviations: B/F/TAF, Bictegravir/Emtricitabine/Tenofovir alafenamide; NRTI, nucleoside/nucleotide reverse‐transcriptase inhibitor; RAMs, resistance‐associated mutations; TAMs, thymidine‐analogue mutations.

^a^ All 4 treatment‐emergent NRTI mutations occurred in participants with virological failure while 2 of 3 treatment‐emergent INSTI mutations occurred in participants with unconfirmed viraemia (single VL ≥200 copies/mL).

After a median follow‐up of 2.4 (IQR 1.3–3.3) years, 188 participants remained in care, 10 had disengaged from care and 8 had died; 153 (74%) had sustained virological suppression, 11 (5%) had unconfirmed viraemia and 43 (21%) experienced virological failure; virological outcomes by ART status at B/F/TAF initiation are shown in Table S2. Of the 54 participants with viraemia on B/F/TAF, 29 (54%) resuppressed while remaining on or restarting B/F/TAF, 5 resuppressed on other ART regimens, 10 remained viraemic on B/F/TAF, 8 remained viraemic on other ART and 2 stopped ART and disengaged from care.

Participants with low CD4 cell counts and/or detectable HIV RNA at B/F/TAF initiation were less likely to achieve sustained virological suppression, while those with NRTI or integrase RAMs appeared to be as likely as those without such RAMs to achieve sustained virological suppression (Table 2). In univariable analysis, the odds of achieving sustained virological suppression were substantially reduced for participants with CD4 < 200 cells/mm^3^ (OR 0.19 [95%CI 0.09–0.38]) or HIV RNA ≥200 copies/mL (0.15 [0.07–0.29]), and those who had interrupted ART (0.25 [0.10–0.62]) at B/F/TAF initiation; these associations were largely unchanged when adjusted for demographic characteristics (0.15 [0.07–0.33], 0.17 [0.08–0.34] and 0.25 [0.10–0.65]). Historical NRTI, INSTI, NNRTI or PI RAMs did not adversely impact virological suppression (adjusted OR 1.28–2.38, *p*‐value 0.20–0.50) (Table S3).

Of the 54 participants with persistent or transient viraemia on B/F/TAF, genotypic resistance data were available for 34 (integrase: 32/34, reverse‐transcriptase: 33/34); 3 (9%) showed novel INSTI accessory mutations (E157Q, L74LM) and 4 (12%) not previously documented NRTI RAMs (M184V/I, D67DN, Y115F) (Tables 2 and S4). Two of those with novel INSTI RAMs had no prior INSTI exposure, while the ART history was incomplete for the third participant; two had a single detectable HIV viral load, and all three re‐suppressed on B/F/TAF. Of those with novel NRTI RAMs, three had NNRTI RAMs, none had PI RAMs and one had the M184V mutation prior to starting B/F/TAF; two re‐suppressed on B/F/TAF, while two restarted B/F/TAF but then disengaged without repeat viral load testing.

## DISCUSSION

In this cohort of people with prior exposure to DRV, B/F/TAF achieved sustained virological suppression in 74%. Baseline viraemia and low CD4 cell count, reflecting ART interruptions or suboptimal adherence to ART, were strong predictors of not achieving sustained virological suppression on B/F/TAF, while historical NRTI or INSTI resistance did not associate with this outcome. Treatment‐emergent accessory INSTI mutations and previously undocumented NRTI RAMs were observed in 9%–12% of those with viral rebound or virological failure on B/F/TAF for whom resistance test results were available. These RAMs did not preclude re‐suppression of HIV RNA with continued use of B/F/TAF, underscoring the importance of adherence to achieving and maintaining sustained viral suppression.

Our findings align with both clinical trial and real‐world data demonstrating the robustness of B/F/TAF, even in individuals with archived NRTI resistance, underscoring the regimen's high genetic barrier to resistance [5, 6, 7, 9, 10, 11]. Treatment‐emergent RAMs were reported in 8 (4.2%) of 189 individuals with virological failure on B/F/TAF in a retrospective analysis of a national French database of genotypic resistance assays; six of these were M184V/I and two were INSTI RAMs (E138K and Y143C emerged in one person each, both with prior exposure to a first generation INSTI), and all had HIV RNA <1000 copies/mL at the time of virological failure [8]. Case reports have described the development of INSTI and NRTI RAMs, mostly in the setting of poor adherence, high baseline viraemia and/or severe immunodeficiency with opportunistic infections [13, 14, 15, 16, 17, 18]. Although none had documented INSTI RAMs prior to starting B/F/TAF, several had a history of virological failure on INSTI‐based ART. Our results add to this experience by highlighting that most individuals with complex resistance can achieve sustained viral suppression on B/F/TAF, that a single episode of rebound viraemia may be sufficient to select for INSTI RAMs, and that such RAMs may be under‐ascertained if not routinely tested for in individuals with unconfirmed viraemia.

Our South London cohort highlights the challenge of achieving sustained viral suppression in a subset of people with HIV. Substantial resources are directed towards maintaining individuals experiencing adherence challenges in care and supporting them in becoming undetectable, including the provision of single‐tablet regimens, intensive adherence support and support to mitigate socioeconomic deprivation and stigma. Nevertheless, 10 (5%) participants disengaged from care and 8 (4%) died during the relatively short follow‐up. These numbers must be interpreted with caution, as those most at risk of not achieving sustained viral suppression may have been maintained on Darunavir‐based ART, and of the 252 individuals who were offered B/F/TAF, 17 (7%) were excluded for not having started B/F/TAF (most did not attend to collect their medication) or lack of viral load measurements on B/F/TAF. The importance of adherence for sustained viral suppression is underscored by the high proportion of participants with viraemia who resuppressed while remaining or restarting B/F/TAF.

This study has some limitations. Its retrospective design, incomplete historical genotype data and single‐centre setting limit the generalizability of our findings. Reasons for the choice of B/F/TAF beyond the change in commissioning policy, adherence to B/F/TAF and the use of concomitant medications that could result in drug‐drug interactions were incompletely recorded and not available for analysis. Moreover, only some participants with viraemia underwent resistance testing. Despite these limitations, the study provides important real‐world evidence that B/F/TAF is an effective simplification option for many individuals who were previously managed on DRV, even in the presence of archived resistance. However, caution is warranted when B/F/TAF does not result in sustained viral suppression, in which case reverting to a boosted PI may help protect INSTIs for future use. It remains unclear how frequently genotyping should be repeated in those who remain viraemic on B/F/TAF to prevent the progressive accumulation of INSTI RAMs.

In summary, we report the emergence of INSTI and NRTI RAMs in people who switched from DRV‐based ART to B/F/TAF. While no treatment‐limiting resistance emerged, continued use of B/F/TAF in the setting of virological failure may predispose to the selection of additional RAMs that could affect the virological efficacy of this regimen. DRV‐based ART regimens remain an essential treatment modality for individuals with complex adherence challenges.

## CONFLICT OF INTEREST STATEMENT

FAP has received grant funding from Gilead, ViiV and MSD (to King's College Hospital), and honoraria or support to attend conferences from Gilead and MSD. All others: no conflict.

## Supporting information


**Figure S1.** Study population.
**Table S1.**Case narratives of the two participants with major INSTI RAMs.
**Table S2.**Virological outcomes by ART status at B/F/TAF initiation.
**Table S3.**Odds of achieving sustained viral suppression on B/F/TAF.
**Table S4.**Clinical characteristics of the participants with novel resistance‐associated mutations.

## Data Availability

The data that support the findings of this study are available from the corresponding author upon reasonable request.

## References

[1] Waters L , Winston A , Reeves I , et al. BHIVA guidelines on antiretroviral treatment for adults living with HIV‐1 2022. HIV Med. 2022;23(Suppl 5):3‐115. 10.1111/hiv.13446 36504313

[2] Paton NI , Musaazi J , Kityo C , et al. Efficacy and safety of dolutegravir or darunavir in combination with lamivudine plus either zidovudine or tenofovir for second‐line treatment of HIV infection (NADIA): week 96 results from a prospective, multicentre, open‐label, factorial, randomised, non‐inferiority trial. Lancet HIV. 2022;9(6):e381‐e393. 35460601 10.1016/S2352-3018(22)00092-3

[3] Aboud M , Kaplan R , Lombaard J , et al. Dolutegravir versus ritonavir‐boosted lopinavir both with dual nucleoside reverse transcriptase inhibitor therapy in adults with HIV‐1 infection in whom first‐line therapy has failed (DAWNING): an open‐label, non‐inferiority, phase 3b trial. Lancet Infect Dis. 2019;19(3):253‐264. 30732940 10.1016/S1473-3099(19)30036-2

[4] Sax PE , Arribas JR , Orkin C , et al. Bictegravir/emtricitabine/tenofovir alafenamide as initial treatment for HIV‐1: five‐year follow‐up from two randomized trials. EClinMed. 2023;59:101991. 10.1016/j.eclinm.2023.101991 PMC10186485 37200995

[5] Behrens GMN , Assoumou L , Liegeon G , et al. Integrase versus protease inhibitor therapy in advanced HIV disease (LAPTOP): a multicountry, randomised, open‐label, non‐inferiority trial. Lancet Infect Dis. 2025:S1473‐3099(25)00681‐4. 10.1016/S1473-3099(25)00681-4 41344354

[6] Trottier B , Yang CJ , Watanabe D , et al. Bictegravir/emtricitabine/tenofovir alafenamide in clinical practice for people with HIV: final 24‐month effectiveness and safety outcomes in key populations in the observational BICSTaR cohort. HIV Res Clin Pract. 2025;26(1):2456890. 39936702 10.1080/25787489.2025.2456890

[7] de Gea‐Grela A , Mican R , de Miguel R , et al. Three‐year effectiveness of Bictegravir/emtricitabine/tenofovir alafenamide as a switch strategy in the real world. Open Forum Infect Dis. 2025;12(3):ofaf087. 40059977 10.1093/ofid/ofaf087 PMC11886859

[8] Marcelin AG , Soulie C , Wirden M , et al. Emergent resistance‐associated mutations at first‐ or second‐line HIV‐1 virologic failure with second‐generation InSTIs in two‐ and three‐drug regimens: the Virostar‐1 study. J Antimicrob Chemother. 2025;80(1):95‐101. 39436753 10.1093/jac/dkae377 PMC11695916

[9] Iwuji C , Waters L , Milinkovic A , et al. Outcomes of switching from protease inhibitor‐based antiretroviral therapy to bictegravir/emtricitabine/tenofovir alafenamide (B/F/TAF) in virologically suppressed adults with nucleos(t)ide analogue resistance‐ a phase IV randomised, open‐label study (PIBIK study). Virol J. 2025;22(1):33. 39930490 10.1186/s12985-025-02648-3 PMC11808997

[10] D'Antoni ML , Boopathy AV , Andreatta K , et al. Brief report: HIV‐1 resistance analysis of participants with HIV‐1 and hepatitis B receiving Bictegravir/emtricitabine/tenofovir alafenamide or dolutegravir plus emtricitabine/tenofovir disoproxil fumarate through the open‐label extension of the ALLIANCE study. J Acquir Immune Defic Syndr. 2025;100(4):342‐346. 40857111 10.1097/QAI.0000000000003749

[11] Shafran SD , Hughes CA . Bictegravir/emtricitabine/tenofovir alafenamide in patients with genotypic NRTI resistance. HIV Med. 2023;24(3):361‐365. 35973753 10.1111/hiv.13376

[12] HIVdb Program: Mutations Analysis Available from: https://hivdb.stanford.edu/hivdb/by-patterns/.

[13] Buzon‐Martin L , Navarro‐San Francisco C , Fernandez‐Regueras M , Sanchez‐Gomez L . Integrase strand transfer inhibitor resistance mediated by R263K plus E157Q in a patient with HIV infection treated with bictegravir/tenofovir alafenamide/emtricitabine: case report and review of the literature. J Antimicrob Chemother. 2024;79(5):1153‐1156. 38558010 10.1093/jac/dkae085

[14] Chamberlain N , Mena L , Brock JB . Case report: emergent resistance in a treatment‐naive person with human immunodeficiency virus under Bictegravir‐based therapy. Open Forum Infect Dis. 2021;8(6):ofab297. 34189182 10.1093/ofid/ofab297 PMC8231367

[15] DeKoven S , Naccarato M , Brumme CJ , Tan DHS . Treatment‐emergent reverse transcriptase resistance during antiretroviral therapy with bictegravir, tenofovir alafenamide, and emtricitabine: a case series. HIV Med. 2023;24(11):1137‐1143. 37317505 10.1111/hiv.13520

[16] Lozano AB , Chueca N , de Salazar A , et al. Failure to bictegravir and development of resistance mutations in an antiretroviral‐experienced patient. Antiviral Res. 2020;179:104717. 31982483 10.1016/j.antiviral.2020.104717

[17] Andre‐Garnier E , Hingrat QL , Marcelin AG , et al. Previously unreported emergence of A265V substitution in the integrase gene in association with bictegravir virological failure. Int J Antimicrob Agents. 2020;56(2):106039. 32479891 10.1016/j.ijantimicag.2020.106039

[18] Rolle CP , Castano J , Nguyen V , Hinestrosa F , DeJesus E . Efficacy, safety, and tolerability of switching from Bictegravir/emtricitabine/tenofovir alafenamide to dolutegravir/lamivudine among adults with Virologically suppressed HIV: the DYAD study. Open Forum Infect Dis. 2024;11(10):ofae560. 39416993 10.1093/ofid/ofae560 PMC11482008

