# Supplementary material for: Virological outcomes with Bictegravir/Emtricitabine/Tenofovir alafenamide (B/F/TAF) in people previously treated with darunavir‐based antiretroviral therapy
Source: HIV Med. 2026 Feb 1;27(5):803–8. doi: 10.1111/hiv.70204 (PMC13140000; doi:10.1111/hiv.70204)
Supplement: Supplementary file 1 — Figure S1. Study population. Table S1.Case narratives of the two participants with major INSTI RAMs. Table S2.Virological outcomes by ART status at B/F/TAF initiation. Table S3.Odds of achieving sustained viral suppression on B/F/TAF. Table S4.Clinical characteristics of the participants with novel resistance‐associated mutations. [file HIV-27-803-s001.docx]

**Fig S1 Study population**


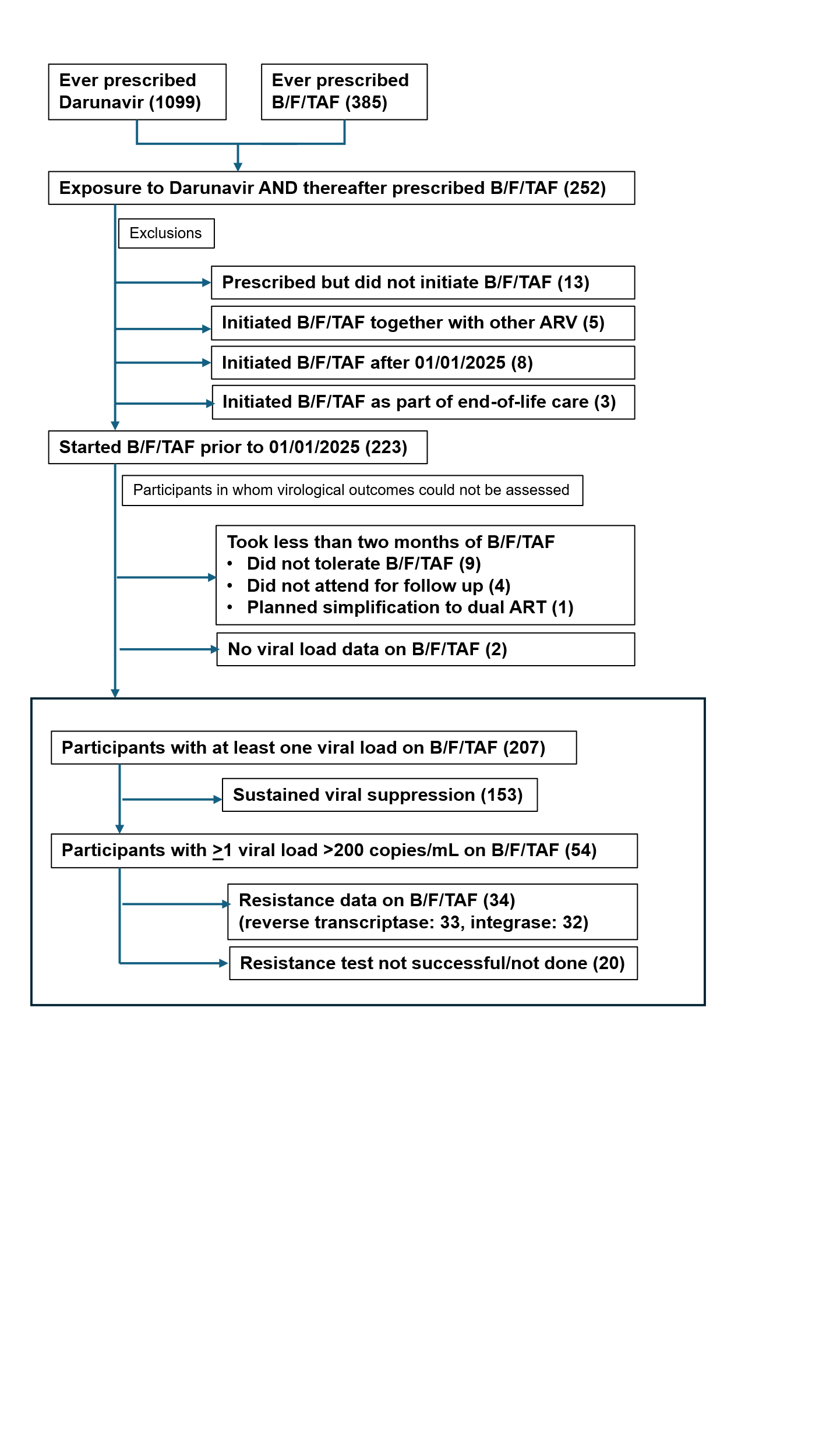


**Table S1 Case narratives of the two participants with major INSTI RAMs**

Participant 1 with K148KQ, 3 TAMs and M184V switched from Dolutegravir/F/TAF to B/F/TAF with an undetectable viral load and maintained viral suppression (mostly VL <20 copies/mL, with two viral blips – VL 65 in 2020 and VL 53 in 2022) to date (six years since switching to B/F/TAF).

Participant 2 with N155H and M184V switched from Darunavir/cobicistat/F/TAF to B/F/TAF with a viral load of 2711 copies/mL and achieved a viral load of 85 copies/mL after six months despite suboptimal engagement and adherence at which point she reported being pregnant and was switched to Darunavir/ritonavir/TDF/FTC and lost to follow up shortly after.

**Table S2 Virological outcomes by ART status at B/F/TAF initiation**

|  | **Sustained VS**** | **Unconfirmed VL >200** | **Virological failure** | **NRTI RAMs*** | **INSTI RAMs*** |
| --- | --- | --- | --- | --- | --- |
| Direct switches from PI to B/F/TAF (N=149) | 117 (79%) | 9 (6%) | 23 (15%) | 2/20 (10%) | 3/19 (16%) |
| Switches from non-PI regimens (N=35) | 25 (71%) | 2 (6%) | 8 (23%) | 0 | 0 |
| ART restarters (N=23) | 11 (48%) | 0 | 12 (52%) | 2/7 (29%) | 0 |

VS=viral suppression (<200 copies/mL); VL=viral load; NRTI=nucleoside/nucleotide reverse transcriptase inhibitor; INSTI=integrase strand-transfer inhibitor; RAMs=resistance-associated mutations

* N with treatment emergent RAMs / N with resistance data

** At the last visit on B/F/TAF, 106 of 117 (91%), 24 of 25 (96%) and 10 of 11 (91%) with sustained viral suppression had HIV RNA <50 copies/mL

**Table S3 Odds of achieving sustained viral suppression on B/F/TAF**

|  | **Univariable analysis** | | |  | **Demographically-adjusted*** | | | | |  |  |
| --- | --- | --- | --- | --- | --- | --- | --- | --- | --- | --- | --- |
|  | **OR** | **95%CI** | **p-value** |  | **OR** | **95%CI** | | **p-value** | | |  |
| CD4 cell count <200 cells/mm^3^ | 0.19 | 0.09-0.38 | <0.001 |  | 0.15 | | 0.07-0.33 | | <0.001 | | |
| HIV RNA >200 copies/mL | 0.15 | 0.07-0.29 | <0.001 |  | 0.17 | | 0.08-0.34 | | <0.001 | | |
| NRTI-resistance | 1.34 | 0.67-2.71 | 0.40 |  | 1.37 | | 0.66-2.83 | | 0.40 | | |
| INSTI resistance | 1.60 | 0.28-9.10 | 0.60 |  | 2.38 | | 0.34-16.8 | | 0.38 | | |
| NNRTI resistance | 1.45 | 0.74-2.85 | 0.23 |  | 1.59 | | 0.79-3.23 | | 0.20 | | |
| PI resistance | 1.30 | 0.65-2.62 | 0.46 |  | 1.28 | | 0.62-2.64 | | 0.50 | | |
| ART status at start B/F/TAF |  |  |  |  |  | |  | |  | | |
| PI-based regimen | ref |  |  |  | ref | |  | |  | | |
| PI-sparing regimen | 0.68 | (0.27, 1.56) | 0.37 |  | 0.72 | | (0.30, 1.75) | | 0.47 | | |
| Off ART | 0.25 | (0.10, 0.62) | 0.003 |  | 0.25 | | (0.10, 0.65) | | 0.005 | | |
|  |  |  |  |  |  | |  | |  | | |

* Adjusted for age, sex and ethnicity

NRTI=nucleoside/nucleotide reverse transcriptase inhibitor; INSTI=integrase strand-transfer inhibitor;

PI=protease inhibitor; ART=antiretroviral therapy; B/F/TAF=Bictegravir/Emtricitabine/Tenofovir alafenamide

**Table S4 Clinical characteristics of the participants with novel resistance-associated mutations**

|  |  |  |  |  |  |  | **At B/F/TAF start** | |  | **Resistance prior to B/F/TAF** | |  |
| --- | --- | --- | --- | --- | --- | --- | --- | --- | --- | --- | --- | --- |
| **#** | **Gender** | **Age** | **Ethnic Group** | **prior INSTI?** | **prior INSTI failure?** | **ART** | **CD4 (cells/mm^3^)** | **VL (copies/mL)** | **HIV Subtype** | **NRTI** | **INSTI** |  |
|  |  |  |  |  |  |  |  |  |  |  |  |  |
| 1 | Female | 51 | Black African | Unknown | Unknown | D/C/F/TAF | 292 | 14,006 | G | M184V | N/A |  |
| 2 | Male | 19 | Black African | No | N/A | D/C/F/TAF | 659 | 93 | A1 | Wild Type | Wild Type |  |
| 3 | Female | 21 | Black African | No | N/A | D/C/F/TAF | 585 | 287 | CRF02_AG | Wild Type | Wild Type |  |
| 4 | Female | 27 | Black British | No | N/A | D/C/F/TAF | 202 | 16,309 | C | Wild Type | Wild Type |  |
| 5 | Male | 62 | Mixed | Yes | Yes | DTG/ABC/3TC | 457 | 29,477 | B | Wild Type | Wild Type |  |
| 6 | Male | 46 | White | Yes | Yes | DTG/F/TAF | 102 | 414,178 | B | Wild Type | Wild Type |  |

|  |  | **At RT** | | **Resistance on B/F/TAF** | | **Clinical course after RT** | |
| --- | --- | --- | --- | --- | --- | --- | --- |
| **#** | **VL pattern on B/F/TAF** | **Time (m) on B/F/TAF** | **VL (copies/mL)** | **NRTI** | **INSTI** | **ART** | **VL (copies/mL)** |
|  |  |  |  |  |  |  |  |
| 1 | 2x VL rebound | 33 | 51,778 | D67DN | L74LM | Restarted B/F/TAF | Re-suppressed to <200 |
| 2 | Single VL rebound | 19 | 120,639 | Wild Type | E157Q | Restarted B/F/TAF | Re-suppressed to <20 |
| 3 | Single VL rebound | 15 | 1,151 | Wild Type | E157Q | Restarted B/F/TAF | Re-suppressed to <20 |
| 4 | Intermittent controlled/uncontrolled | 20 | 5,864 | M184V | Wild Type | Restarted B/F/TAF | Re-suppressed to <20 |
| 5 | Single VL rebound | 17 | 514,837 | M184I | Wild Type | Restarted B/F/TAF | Disengaged/no repeat VL |
| 6 | Persistent viraemia >100,000 | 28 | 1,965,358 | Y115F | Wild Type | Restarted B/F/TAF | Disengaged/no repeat VL |

INSTI=integrase strand-transfer inhibitor; ART=antiretroviral therapy; D/C/F/TAF=Darunavir/Cobicistat/Emtricitabine/Tenofovir alafenamide; VL=viral load;

NRTI=nucleoside/nucleotide reverse transcriptase inhibitor; B/F/TAF=Bictegravir/Emtricitabine/Tenofovir alafenamide; RT=resistance test
